# Advance care planning with older Norwegian adults in their homes: a narrative ethnographic study

**DOI:** 10.1186/s12904-024-01378-7

**Published:** 2024-02-19

**Authors:** Line Elida Festvåg, Beate Lie Sverre, Ørnulf Paulsen, Grethe Eilertsen

**Affiliations:** 1 USN Research Group of Older Peoples’ Health, University of South-Eastern Norway, Drammen, Norway; 2 Department of Nursing and Health Sciences, Faculty of Health and Social Sciences, University of South-Eastern Norway, Drammen, Norway; 3 Palliative Care Unit, Telemark Hospital Trust, Skien, Norway; 4 Dept. of Oncology, European Palliative Care Research Centre (PRC), Oslo University Hospital, Oslo, Norway

**Keywords:** Advance care planning, Older, Relatives, Home, Palliative care, Narrative ethnography

## Abstract

**Background:**

The aim of advance care planning (ACP) is to enable patients to define and discuss their values and preferences to ensure that the care they receive is consistent with their needs and wishes. Most studies of ACP with older adults focus on conversations conducted in institutions. This study aimed to explore how ACP with older patients is carried out and experienced by healthcare professionals when the conversations occur in their private homes.

**Methods:**

The data were obtained from participant observations of ACP conversations in the homes of eight older patients with advanced cancer, which also involved relatives and healthcare professionals. Additionally, ethnographic interviews were conducted with the healthcare professionals. We undertook a narrative analysis of what was said, and how the individuals acted and interacted.

**Results:**

The home influenced both the substance and form of the ACP conversations. The patients and relatives welcomed the healthcare professionals as guests and were encouraged to share their perceptions of their current situation, joys and worries. Their values were often implicit in their stories about past experiences. The planning mainly focused on life-prolonging treatment and the preferred future place of care. Several patients were not ready to discuss one or more ACP issues. The palliative-care-team physician addressed the patients’ readiness for ACP by asking for permission to move on to a different topic, shifting between serious and lighter topics, and using elements from the home as ‘door openers’ to continue conversations. ACP conversations were an essential basis for future palliative care and cooperation, giving important additional information about the patient and their relatives.

**Conclusion:**

Conducting the ACP conversations in the patients’ homes ensured a homely atmosphere that facilitated a caring approach when sensitive issues were discussed, and in turn supported the identification of important personal values. The healthcare professionals expressed that the ACP conversations represented an essential common reference point and provided a shared awareness of the expected disease trajectory and the values, preferences and needs of the patient. These findings are particularly important given that many older patients struggle to verbalize or form an opinion on issues affecting their future.

## Background

Most patients with advanced cancer have extensive care demands and want to participate in planning around their care to ensure that the decisions made align with their wishes [1]. However, studies [2–4] have shown that older adults only occasionally participate in conversations about their future care. To facilitate both the spent time at home and dying in the home, advance care planning (ACP) is encouraged in the patient palliative-care trajectory in Norway [5]. ACP is a communication process that enables patients to identify their values, reflect upon issues such as the meanings and consequences of a serious disease, and address their concerns across physical, psychological, social and spiritual domains [6]. Furthermore, ACP aims to define and discuss goals and preferences for future medical treatment and care [6]. In a recent meta-analysis, Wang et al. [7] found that ACP improves the end of life of older adults. Timely documentation of the preferred places of care and death may result in less healthcare consumption and a better match between the preferred and actual places of death among older patients with cancer [4]. However, ACP conversations generally have low uptake, and primarily occur late in the disease trajectory [8].

Most patients want to live and die at home [9]. Particularly among older patients, their home reminds them of who they are, surrounded by their belongings and relatives, providing emotional support and security. Living in their home also allows older patients to carry out their familiar daily activities for as long as possible [10, 11]. Home-based palliative care reduces the symptom burden, improves the quality of life and increases the probability of dying at home [12, 13]. The older person’s relatives contribute significantly to the quality of home-based palliative care [14]. However, they reportedly often feel unprepared to perform caregiving tasks, experiencing a heavy burden that impacts both their emotional and physical health [15–17]. Including relatives when developing and revising care plans might therefore increase their satisfaction with the care delivered to patients and better prepare them for the terminal phase [14, 18].

ACP in end-of-life care has been studied widely [19], but only a few studies have observed participants during ACP conversations. Andreassen et al. [20] found that patients and relatives were encouraged to reflect upon and make informed choices during ACP conversations. The patients actively asked questions about their current situation, but some of them challenged the concept of ACP and the ability to take control of end-of-life issues in advance. In a study conducted in nursing homes, Thoresen et al. [21] found that patients were quiet, passive and apprehensive when discussing ACP issues. Those authors also found that while the healthcare professionals aimed to support open awareness, autonomy and a good death, this was often not achieved; in follow-up interviews, there was little reflection on the purpose, issues and timing of ACP, or on the capacity of frail nursing-home patients to comprehend and communicate during ACP conversations. Sævareid et al. [22] reported that residents with cognitive impairment in nursing homes actively and meaningfully participated when the healthcare professionals actively listened. Andrews [23] identified that ACP was applied in a disjointed manner, in which relationships and management were disrupted by the structure and organization of care, resulting in the fragmentation of decision-making and marginalization of the patients’ voices. In a study of home-based ACP, Walshe [24] found that physical symptom relief was emphasized, while planning for future palliative care was somewhat overlooked. Based on the insight provided by these observational studies, we considered that participant observations combined with follow-up interviews would reveal useful information about how the home context affects ACP conversations.

Thus, this study aimed to explore how ACP is carried out and experienced by healthcare professionals when performed in the homes of older patients. The research questions (RQs) were as follows: (i) how do healthcare professionals prepare themselves and the patients for ACP conversations, (ii) how is ACP conducted in the homes of patients, and (iii) what is the significance of ACP from the perspective of healthcare professionals? The present study was the first part of a research project aiming to understand the perspectives of older patients, relatives and healthcare professionals on the significance of ACP when carried out in the homes of patients.

## Methods

### Design

A narrative ethnographic approach was used in this study to capture the complexity of ACP. Ethnography is a qualitative, explorative research methodology used to develop descriptions [25] of social interactions, events, behaviours and perceptions [26]. This approach makes it possible to grasp complex contexts and study broader, contextual aspects of significance [27]. The narrative approach involves constructing narratives based on ethnographic descriptions, and these narratives serving as the unit of analysis [28]. Narrative ethnography analyses stories in their context [29]. The design chosen in the present study is highly consistent with a recent call for ethnographic and narrative research of ACP to respectively gain a better understanding of (ii) how practice and experiences are shaped within a specific context, and (ii) the meaning-making of ACP through the storytelling of their particular experiences [30].

### Setting

Patients with cancer in Norway are transferred to municipal palliative-care services when their anticancer therapies are discontinued. Municipal cancer nurses (hereafter, cancer nurses), home-based care services and the general practitioner (GP) provide palliative care in the patient’s home, while the hospital-based palliative-care team (PCT) provides specialist advice.

**Fig. 1:**
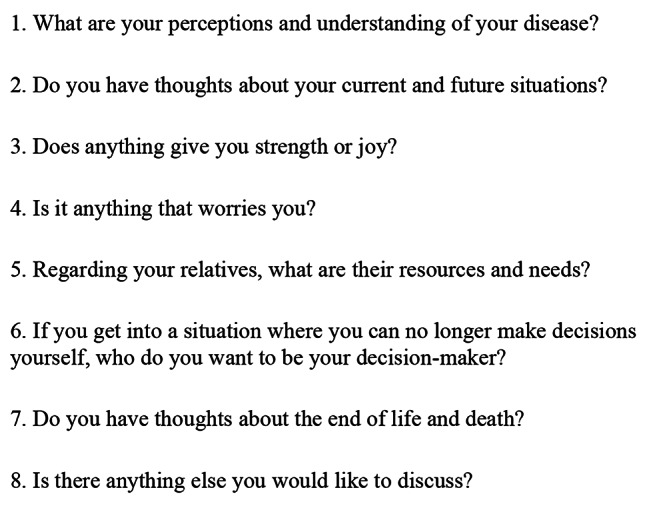
Questions related to ACP issues in the information brochure

Upon referral and shortly after anticancer treatment has ended, a palliative-care coordination meeting is held with the patient in their home together with their relatives, the cancer nurse, GP and PCT. The ACP conversations observed in this study took place as part of these meetings. A patient information brochure with questions related to ACP issues was given to the patients before the meetings (Fig. 1).

### Recruitment and sample

The PCT and cancer nurses screened eligible patients ≥ 65 years old with advanced cancer living at home who had recently stopped or were not eligible for anticancer therapy and had a score on the ECOG Performance Status Scale of ≤ 3 [31] (Table 1).

**Table 1:** Inclusion and exclusion criteria

Inclusion criterion	
Age	≥ 65 years
Gender	Male or female
Medical care	Referred to PCT
Living situation	In their own home
Residence location	Urban or rural
ACP at home	Agreeing to participate in ACP conversations with a cancer nurse, GP and PCT
Language	Fluent in written and spoken Norwegian
Consent	Able to give informed consent to participate
Relatives	Patient agreed to eligible relatives being invited to participate
**Exclusion criterion**	
ECOG Performance Status Scale score of 4	Patients assessed as completely disabled or confined to a bed or chair

The patients and their relatives received both verbal and written information about the study. The patients needed to consent to the participation of their relatives. The cancer nurses relayed the oral consent to the PCT, who recruited the GPs and cancer nurses. Consent from all participants was collected before any study procedures were performed. The study sample comprised eight patients from one Norwegian county (Table 2). Additionally, participants present during the ACP conversations were 15 relatives, six GPs, eight cancer nurses and the PCT (consisting of one nurse and one physician) (Table 3). All names used in the result are fictitious.

**Table 2:** Patient characteristics at the time of the ACP conversations

Patient	Age, years	Cancer diagnosis	Time since diagnosis	Time since anticancer therapy ended	ECOG score	Living condition	Home-care services
Alf	79	Lung CA	2 years	6 weeks	1	Lived alone. Son and daughter nearby	3–4 times/day
Birger	88	Prostate CA	11 years	3 years	1	Lived alone. Daughter nearby	2 per day
Catherine	83	Colon CA	1 year	8 weeks	1	Lived with husband. Daughters nearby and far away	None
Didrik	78	Prostate CA	17 years	13 weeks	2	Lived with wife. No children	Once a day
Else	82	Pancreatic CA	3 months	Not received	3	Lived with husband. Two daughters nearby	4 daily
Finn	71	Renal CA	2 years	2 weeks	3	Lived with wife and daughter	3 daily
Guri	92	Leucaemia	2 months	Not received	3	Lived with husband. Sons and daughter nearby	Once a week
Hannah	82	Uterine CA	2 years	9 months	3	Lived alone. Daughter nearby	Once a day

**Table 3:** Participants present during the ACP conversations

Patient	Spouse	Adult children		Cancer nurse	GP	PCT: physician & nurse		Researcher
		Son	Daughter(s)					
Alf	-	Amund	Astrid	x	x	x	x	x
Birger	-		Bjørg	x		x	x	x
Catherine	Chris		Camilla Cate	x	x	x	x	x
Didrik	Dina		-	x	x	x		x
Else	Einar		Elise Elin	x	x	x	x	x
Finn	Frida		Freja	x	x	x		x
Guri	Gunnar	Glen		x		x	x	x
Hannah	-		Hege	x	x	x	x	x

### Data generation

The ethnographic fieldwork involving participant observations and ethnographic interviews was conducted between October 2021 and October 2022. The fieldwork consisted of (i) ethnographic interviews with the PCT before the ACP conversation (to address RQ 1) (interview guide as additional file 1), (ii) participant observations of the ACP conversation (to address RQ 2) (observation guide as additional file 2) and (iii) ethnographic interviews with the PCT, GPs and cancer nurses immediately after the ACP conversation (to address RQ 3) (interview guide as additional file 3). Field notes were taken during each ACP conversation, and reflection notes were written thereafter. The first author audio recorded the interviews and ACP conversations, and then transcribed them verbatim. The mean durations of the different components of the fieldwork were as follows: the interview with the PCT before the ACP conversation took 20 min, the observation of the ACP conversation took 60 min and the interview with the healthcare professional after the ACP conversation took 30 min.

### Data analysis

Narrative ethnography focuses on the *how* (the external organization) and the *what* (the internal organization of the narrative) [28]. The analysis was applied to both the descriptions of the context in which ACP was carried out (external conditions) and what was discussed (the content). Data analysis was performed simultaneously with data generation. Each data set with transcripts and field notes from one ACP informed the other, in an iterative process.

A thematic, structural and performative narrative analysis [32] was conducted to develop theoretical reasoning. The material was read thoroughly with guidance from the sensitizing RQs. The different data sources were scrutinized for each ACP conversion and then compared across the eight ACP conversions in order to identify overarching themes and patterns, differences and similarities, as well as how the participants acted and interacted. A structural analysis subsequently applied ACP as a communication process. A narrative of how ACP was carried out and experienced by healthcare professionals was constructed as events organized along a timeline with a start, middle and end.

The narrative below includes the participants’ voices from both ethnographic interviews and participant observations with the intention of keeping and conveying the vividness and authenticity of these voices. The quotes encapsulating the core findings have been shortened, cleaned of dialect and filler words, and italicized. The researcher’s observations are labelled as field notes. Relatives are labelled according to their connection to the patient. The term ‘physician’ is used when referring to the physician in the PCT.

### Ethical considerations

Older adults in the palliative phase are considered vulnerable [33], and so extra care was taken to ensure that the health of the included patients remained relatively stable. Informed consent was obtained from all participants at the beginning of each interview and ACP conversation. Approval according to Norwegian research law and regulations was obtained for this study from the Norwegian Agency for Shared Services in Education and Research (Reg. no. 316723).

## Results

The constructed narrative of how ACP was carried out in the home context commenced with preparing for the ACP conversations. After arriving at the home, the conversations about sensitive topics were facilitated by the homely atmosphere. After the conversations ended, the healthcare professionals considered their experiences of the conversations.

### Planning for continuity and flexibility

To prepare for each ACP conversation, the PCT gathered information about the patient’s health situation and expected disease trajectory. This served as a foundation for maintaining continuity with prior agreements during the conversation. While she sat in her hospital office, the PCT nurse’s story captured the essence of their preparation:

*We collaborate with the hospital oncologist, the cancer nurse and the GP to obtain an overview of the diagnosis, the anticancer therapy and the reason for stopping the treatment. We coordinate with the GP and the cancer nurse to better understand the patient and their relatives, and schedule a time for the coordination meeting and ACP conversation. The cancer nurse ensures that any changes in the patient’s situation before the ACP conversations are reported to us. The patient and relatives are informed orally and given the information brochure either by the oncologist before discharge from the hospital or by the cancer nurse.*

The physician emphasized the need to be flexible when meeting the patients:

*It’s important not to push on issues they are not ready for. We let them come along with what they want to tell us and then move naturally to more serious topics.*

### Welcomed as guests

The following observation from Catherine’s home describes a typical welcome scene:

Chris (her husband) opened the door, smiled and bowed slightly as he put his right arm on his chest, and explained: ‘*In these COVID times, we greet as they do in Asia*’. He graciously asked for our coats before guiding us into the living room, where we were greeted by Catherine, their two daughters (Camilla and Cate), the cancer nurse and the GP. The daughters asked us to take a seat around a coffee table. The family took their places in a semicircle around one end of the table, and the healthcare professionals and I sat at the other. All except the cancer nurse were wearing personal clothes. It was a cold winter, but a woodstove made the temperature indoors comfortable. Catherine exuded a great calmness with her direct, warm gaze and appeared to have an overview of the fact that everyone had taken a seat and had tea or coffee. Everybody complimented her excellent cookies, and Catherine reassured us that she had plenty more. (field notes)

This vignette illustrates that the patients and relatives assumed the role of hosts in this study, welcoming the healthcare professionals as guests. The GP and cancer nurses were familiar to most patients, while the PCT was unfamiliar to all except one of the patients. Some of the small talk was about objects such as photographs and antique furniture. The comments and questions from the healthcare professionals in connection to what they observed aimed to elicit personal stories from the family.

### The home context increases awareness of the patient as a person

Meeting patients in their homes provided information about values and relationships:

*To see the patients at home provides valuable information. A well-kept house, pictures of children and stories about relationships with friends and family give hints about values. Observing how they interact and support each other informs us about relationships within the family.* (PCT nurse)

According to the physician, the home context influences how the patients behave:

*They seem to be more relaxed, which makes it easier to get an impression of who they are, and their resources and needs*.

Values were often not explicitly expressed, but were implicit in stories about past experiences. Birger’s daughter explained how her father cared for people, conveying the importance of having good relationships with others and contributing to their well-being:

*He was occupied with something and someone all the time. When driving the car, he told us exciting and funny stories. He still meets friends every day for dinner*.

Stories about who the patient had been and was now typically started immediately after the healthcare professionals entered the home and continued throughout the conversations. The welcoming scene usually concluded with the physician expressing gratitude for being invited, and the ACP conversations then progressed, with the physician stating their aim to support the patients and their relatives during the upcoming period. The subsequent conversations were led by the physician, and most conversations were between him and the patients.

### Conversations about sensitive topics were facilitated by the homely atmosphere

#### Planning for the future by looking at the past and present

The ACP conversations addressed issues connected to the past, present and near future, and the homely atmosphere seemed to support what was being told. The patients’ previous professional careers, their homes, family lives and experiences with disease and death were common themes. The patients shared (sometimes in detailed narratives) their experiences with diagnosis, anticancer therapy and meetings with healthcare professionals, as well as how they coped with the challenging transition after stopping anticancer therapy. The physician gave supplementary details, summarized their medical history, and encouraged the patient and relatives to ask questions.

The conversations focused on the present status of basic needs and consequences of the disease, such as the presence, intensity and effects of symptoms, as well as possible adjustments to medical treatment. These discussions were primarily between the physician and patients. In all conversations, the patients’ current main challenges were asked about by the physician, with the patients seldom mentioning their concerns unprompted. When asked, Didrik responded: ‘*I go to bed before the pain gets too strong, and hopefully, I fall asleep. Sometimes I do, but then I wake up with the pain*’. Typically, the questions focused on the perceptions of both the patient and relatives about the current situation, joys, worries and religious affiliation.

The relatives supported their patient during each ACP conversation by asking and responding to questions or laying a hand on the patient’s arms or shoulders. In this part of the conversation, the relatives talked about their concerns, confirming the patient’s experiences or adding perspectives that differed from the patient’s view, as the following observations highlight:

Else was diagnosed with pancreatic cancer 3 months before the ACP conversations. When she claimed that she was not eating too little, the physician looked at the relatives and asked: ‘*Are you worried about her decreasing appetite?*’ Her husband nodded, and the youngest daughter (Elise) said: ‘*You know, all she wants is pancakes*’. The physician informed them about decreasing nutrition needs as part of the natural dying process, and the family showed great gratitude and relief at not having to emphasize nutrition. (field notes)

According to the physician, monitoring the relatives will facilitate discussions about any discrepancies or concerns. The relatives maintained a reserved attitude towards their needs during the ACP conversations, except when directly asked about who they were, how they felt and how they looked after their health, especially when they expressed that they were tired. As Dina said, ‘*I sleep less, and my headache is bad. However, my biggest burden is to see him in pain*’. The physician often stated how important self-care was to ensuring that relatives were able to care for their patient. Whether the concerns or resources of the relatives were sought and discussed varied, but in all of the ACP conversations the physician stressed the need to give relatives a sense of security.

At one point in the ACP conversations the focus shifted towards the future. After asking for permission from the patient, the physician provided information about the expected disease trajectory. These discussions included possible treatment options, including whether hospitalization would be necessary or wanted. The patients were encouraged to share their concerns and thoughts about treatment and hospitalization if a life-threatening situation should occur. Some, like Alf, expressed their wish to restrict life-prolonging treatment:

Physician: *You have been referred for radiotherapy, Alf.*

Alf: *Yes, but I want to discuss it with you.* (He picked up a letter from the table.) *I am so tired.*

Physician: *Okay, let’s discuss it. You know, to cancel the planned radiotherapy* *may cause things to progress more quickly.*

Alf: *I can’t take the radiotherapy.*

Physician: Y*ou have made up your mind already, have you?*

Alf: *Yes.*

All patients expressed a fear of receiving futile treatment, and so were assured that no one would be subjected to non-beneficial treatment. When the physician asked about the patients’ wishes concerning future places of care, all but one patient expressed a desire to stay in their home for as long as possible and to die at home; the single exception, Alf, wanted to die in a nursing home for his children’s sake: ‘*I don’t want to burden my children. They must take care of their work and not me*’.

In all of the ACP conversations, the physician emphasized that the medical decisions were the physician’s responsibility. He asked the patients about whether they would allow him to make decisions regarding life-prolonging treatment based on their values and wishes, which they all agreed with. Some patients were asked whether their spiritual needs were met, and they were offered a chaplain service. In addition, future home-care issues were discussed if the patient or their relatives requested more assistance from the home-care services or medical or technical aids.

#### A caring approach

The physician had a prominent role in leading the conversations with the patients and relatives. The cancer nurse, GP, and PCT nurse had a withdrawn role and only occasionally contributed with additional information or elaborative questions and comments. However, they contributed to the development of the conversation through their attentive presence.

The physician takes the floor. He is experienced in coordinating municipal palliative care and ACP and drives the conversations forward with in-depth questions or new ones. The GP, cancer nurse and PCT nurse are focused on what is being said, often maintain eye contact with the person speaking, smile or nod affirmatively, and make supportive comments. (fieldnotes)

Their confirming attitude seemed of particular importance as several patients were ready to share their thoughts.

The ACP information brochure was given to the patients at 2 to 14 days before meeting them in their homes, except for two women who did not receive the brochure. Although all patients accepted the invitation to participate in the ACP conversations, some were reluctant to discuss potential future treatments and end-of-life issues. It seemed difficult for them to imagine future conditions, and most such patients left such decisions to the GP. The ACP conversation with Finn revealed that the physician was familiar with their refusal to discuss the future. Nevertheless, he turned to Finn’s preferences for medical life-prolonging treatment:

Physician: *What matters to us is that your thoughts and desires are reflected in what we do and don’t do.*

Finn (in a weak voice): *It’s hard to imagine the future. I know nothing about it. My GP decides.*

The ease with which patients and relatives discussed their values, preferences and needs varied. At one end of the spectrum was Guri, who did not allow these topics to be addressed:

Guri found it challenging to get used to the situation. Until recently, despite her advanced age, she had a great capacity for work. With a raised voice and more hectic speech, she made it clear that she did not want to talk about what lies ahead, and her husband confirmed: ‘*We always take one day at a time*’. The physician nodded and asked whether she would allow her son to discuss these issues with the PCT without her. Guri consented. (field notes)

Due to ACP involving sensitive issues, it is worth noting how the physician moved cautiously towards more-challenging topics, to assess the patient’s readiness for ACP. Aiming to let the patients decide whether they would allow him to progress, the physician typically asked: ‘*Would you allow me to ask you some more sensitive questions?*’, to which they all consented. The physician also observed emotional expressions such as crying, evasive answers or direct rejection as cues for when to stop. In the interviews after the ACP conversation, he explained: ‘*I aim to prepare the ground for their decisions by listening to and looking at their reactions, and then deciding whether or not to move on. If I push them too hard, it turns brutal*’. The PCT nurse commented: ‘*It requires courage to slightly push while respecting their limits*’.

A typical pattern in all of the ACP conversations was alternating between serious and lighter topics. In addition to addressing medical issues, a common theme across the conversations was the inclusion of what brings joy in the everyday lives of the patients. The following field notes convey the importance of participating in daily life:

Finn looked at the physician, breathed and answered: ‘*My grandson is playing football next week, and I hope to see him playing*’. An energetic discussion followed to find a solution for getting Finn to the football pitch. The conclusion was to organize transportation in a wheelchair.

Humour provided a break from sensitive topics and was present to varying degrees in all of the ACP conversations. This is exemplified in the conversation with Guri:

To Guri, the diagnosis was a shock, and discussing the upcoming blood transfusion was difficult. The physician asked, ‘*Who keeps the house so clean and tidy*?’ Guri laughed heartily and looked at her 96-year-old husband: ‘*Well, he’s not as quick on his feet as before*’. I could feel the relief of laughter, and the alternation between seriousness and banter was fascinating. (field notes)

Elements from the home were typically used as ‘door openers’ to continue conversations. A photograph served this purpose in Birger’s ACP conversation:

In the entrance hall, a photograph of a smiling lady was visible. His face lit up when the physician asked about her: ‘*We were always standing together. Then she got cancer*’. After a short break, the physician asked: ‘*Is there something you experienced then that worries you now?*’ Birger said ‘*No*’ in a clear and distinct voice. (field notes)

The homely atmosphere influenced both the substance and form of the ACP conversations. It also seemed to facilitate discussions in an environment where sensitive issues could be approached positively and in a caring manner.

### Ending the conversations, and how health professionals experienced the conversations

A consistent pattern in all conversations was a clear transition from the central part of the conversation to the end part, initiated by the physician. The following quote is representative of the typical end phrasing:

*All ACP conversations are digitally summarized and made electronically available to you via the web service provided by the Norwegian health authorities, to the home-care service and to the healthcare professionals*. *The municipal healthcare professionals will meet you regularly and represent the first line of contact, and we will be backstage. We will visit you again when necessary.* (physician)

The conversations were not systematically summed up at the end of the ACP conversation. However, a follow-up conversation was mentioned but not exactly planned. Since the GP and cancer nurses were familiar to most patients, agreements on how to proceed with their relations with the patients were already planned. Consequently, “see you next week” or similar were typically farewell comments. Patients and their relatives expressed their gratitude by welcoming the healthcare professionals back.

In the interviews immediately following the ACP conversations, the healthcare professionals expressed that the conversations were essential for coordinating future palliative care and cooperation. One cancer nurse highlighted that this was an opportunity to discuss the issues that patients usually do not discuss, which could make follow-up ACP conversations easier. Furthermore, they highlighted the importance of coordinating the different healthcare professionals so that their relative competencies could be revealed and understood. The meeting was expressed as valuable, representing an essential common reference point and providing a shared awareness of the expected disease trajectory.

## Discussion

This study involved ACP conversations with older patients in their own homes along with their relatives and healthcare professionals. The home context influenced how the participants acted and interacted during the conversations, as well as the content of those conversations. The patients and relatives assumed the role of hosts and welcomed the healthcare professionals as guests. In the physician-led ACP conversations, patients and relatives were invited to share their perceptions of the current situation, joys, and worries. The physician informed the patients and relatives about the expected disease trajectory and possible types of palliative medical treatment.

The patients’ readiness to discuss ACP affected its potential outcomes, and it varied as to the ease with which patients and relatives discussed their values, preferences and needs. While values were often not expressed explicitly, they were implicit in stories about past experiences. The physician moved cautiously towards more-challenging topics. A typical pattern across the conversations was to alternate between serious and easy-to-talk-about topics, and to use humour. Elements from the home were also typically used as ‘door openers’. According to the healthcare professionals, the conversations conducted in the patients’ homes provided valuable information about values and family relationships, and were essential for ensuring optimal future palliative care and cooperation.

### The home context provides valuable information

The use of the home context in this study provided the healthcare professionals with valuable information that might otherwise be difficult to obtain. Being in a familiar context made it easier for patients to reflect on and voice their values, preferences and needs. Small talk seemed to help to establish relationships, and it elicited personal stories, which subsequently highlighted what was at stake for the patients and their relatives, including hints about valued relationships. A home is a place where identities are promoted and expressed socially and culturally [34], and all but two patients had lived in the same house for most of their adult lives. Meeting with patients in their own homes, surrounded by their personal belongings and relatives, allowed healthcare professionals to get closer to the everyday lives and personalities of both the patients and their family members. Palliative care is often associated with a threat to the patient’s identity, which makes it valuable to be able to observe them in a context that strengthens the impression of who they are.

The present analysis revealed the value of observing the relationships and discussions within the families. Only some of them talked openly about their illness and impending death. Family dynamics affect ACP in complex ways [35], and it is essential to understand the patterns of how patients and relatives interact with each other [36]. In the present study, the relatives supported the patients by confirming their experiences, asking and responding to questions, or through physical contact, which was interpreted as a wish to be close to and convey that they were standing together as a unit. This is consistent with previous studies showing that patients view their relatives’ presence as essential to the quality of ACP [37].

### Patients as hosts and professionals as guests

A particularly notable observation in this study was how the patients and their relatives played active roles as hosts when welcoming the healthcare professionals. Accordingly, the healthcare professionals assumed the role of guests when entering their homes. Being in a familiar context during the conversations and meeting healthcare professionals dressed in their personal clothes was seen as having positive impacts. This approach helped to make the patients and their relatives more visible as ordinary people and to alleviate the unpleasant feelings that might be evoked during the ACP conversation. This might also have mitigated the power imbalance between the patients and relatives on one side and the healthcare professionals on the other. Confronting end-of-life issues forces patients to face their imminent death, which can result in them feeling disrupted and distressed [38]. There is a great deal at stake for patients and their families at the end of life, and the stories around the coffee table seem significant beyond just letting the guests know they are welcome. Öresland et al. [39] differentiated between healthcare professionals being guests or professionals when visiting patients at home. They found these two roles appropriate for understanding how healthcare professionals balance between seeing the patient as a subject in the world – which restricts what healthcare professionals can allow themselves to do – and needing to act decisively if required to do so. Those authors interpreted the role of guest as a construction of ‘equality’, which might reduce this power asymmetry. While healthcare professionals in healthcare institutions control the care environment, the reverse is true in patients’ homes [40]. Karlsson et al. [41] considered that the home context contributes to equalizing the unequal power relationships between healthcare professionals and patients.

Performing the ACP conversations in homes in the present study rapidly established a sense of safety for the patient and their relatives. The initial conversation about family hobbies flowed easily, and visible household objects created an open and relaxed atmosphere that was fundamental to facilitating conversations about personal values, preferences and needs. The patient narratives seemed to be more credible and easier for healthcare professionals to access when they were told in the home context. When patients actively assume the role of host and share their experiences of disease and life, they are essentially communicating ‘*This is who I am, and this is the life I’ve lived*’. It is a way of asserting that they should not be reduced to something less than their full selves.

### The content of the conversation and the physician’s caring approach

Observing the conversations revealed how the physician prioritized clarifying the symptom status with both patients and relatives before moving forwards with the conversation. The topics moved from medical and social to the values and personal stories of the patients. The place of care, criteria for hospital admission, issues concerning resuscitation and how they view their current life and future were all central ACP topics. Patients with high symptom burdens were primarily concerned with how healthcare professionals could help to alleviate them; medical issues were consequently prioritized in those conversations. Thoresen et al. [21] critiqued taking clinical and physical approaches in ACP conversations, highlighting the importance of including psychological, social, existential, spiritual and emotional aspects in discussions about end-of-life issues. Our results show that existential issues were addressed in the patients’ narratives about lived life and what the patients found meaningful, and in discussions about life-prolonging treatment. We also found that establishing trust, security and symptom relief seemed to be prerequisites for discussing other topics. Notably, less attention was paid to the needs and resources of the relatives, and when they were talked about, this was brought up by the physician. Borregaard Myrhøj et al. [42] highlighted the importance of ensuring that the concerns and fears of relatives are communicated during ACP conversations. Follow-up conversations focusing on the relatives therefore seem necessary.

The physician underlined the need to be sensitive to the emotions of patients and relatives and to tailor conversations according to the readiness of patients to discuss ACP, which aligns with ACP recommendations [6]. A notable pattern that appeared across all conversations was alternating between serious, disease-related topics and lighter conversations interspersed with humour. This was interpreted as taking a break from the topics that were difficult to talk about. Previous studies have shown that ACP conversations are emotionally charged [32], and humour can serve as a diversion for participants who are uncomfortable discussing severe sickness and death. Furthermore, these exchanges in the present study allowed for flexibility regarding variations in the abilities of patients to discuss urgent issues and contemplate the future. This flexibility seemed particularly important given that the results revealed variations in the ease with which patients talked about complex topics. The physician was sensitive when leading all conversations forwards, which might empower patients and relatives to voice their concerns and choices when discussing sensitive issues [18]. The withdrawn role of the GPs, cancer nurses and PCT nurse may have helped to reduce the possible overwhelming effect of many and unfamiliar healthcare professionals present. Another aspect might be how the highly experienced physician assumed the role of expert and the municipal healthcare professionals valued the potential learning effect by observing him practicing palliative care.

### The necessity of readiness

Some patients had already made well-defined decisions about their future palliative care, whereas others were unable to or chose not to form an opinion, as has been found previously [43, 44]. Moreover, some patients expressed a preference for their relatives and physician to make decisions about life-prolonging treatment. It is reasonable to assume that the readiness for ACP influences the ability and willingness of a patient to discuss their future palliative care. This readiness is affected by the degree to which they are mentally prepared to listen to and understand information, ask questions, and communicate their preferences and wishes [45]. However, older patients may have difficulties understanding the consequences of their decisions [46]. When their life expectancy is short, they might focus on outcomes rather than treatments and not actively engage in decision-making [47].

The present study found that having a clear understanding of what ACP entails and which topics should be discussed influence the experiences of patients as well as the conversation outcomes. Thus, we call for the development of new approaches for healthcare professionals to facilitate the readiness of older patients for ACP, such as by offering patients and relatives conversations *about* ACP in order to increase their readiness.

For some patients, the homely atmosphere seemed to support the caring approach and make it easier to discuss ACP topics. For others, this was not enough. Building trust takes time, and so the motivation of the patients in this study to talk about personal issues may have been influenced by them meeting some of the healthcare professionals for the first time. It is reasonable to assume that talking with many (and new) people in ACP conversations may reduce the openness of participants about personal matters. Since ACP is considered a communication process, the present study only represents a starting point. To address the need for clarification and to cover questions not discussed initially, the patient and relatives should be offered follow-up conversations to ensure the effectiveness of the ACP communication process.

### Strengths and limitations

This study aimed to explore how ACP is carried out and experienced by healthcare professionals when performed in the homes of older patients. A comprehensive approach was applied to obtain an in-depth understanding of how ACP was carried out and experienced with a small number of participants. Using an ethnographic design allowed us to thoroughly investigate what happened, rather than relying on retrospective descriptions. We succeeded in exploring how the participants acted and interacted, thereby generating a detailed understanding of how the home context influenced the ACP conversations. The goal of having a balanced gender distribution and participants from both urban and rural locations was also accomplished.

The interpretation process was constant and carefully discussed by the multi-professional research team to achieve consensus and validate the emerging findings. Thorough descriptions of the study context, analysis strategy and quotes from the empirical material further enhance the trustworthiness of this study.

However, we did not obtain information about the patients who did not want to participate in this project, nor their reasons. Our data mainly reflect positive attitudes towards ACP, with regards to taking part in the conversations and their importance for the follow-up of the patients and families afterwards. It is reasonable to assume that patients with negative attitudes towards ACP would have refused to participate in the study. The presence of both the patients and their relatives in the ACP conversations can be viewed as a strength, since this allowed healthcare professionals to observe the interaction between these two parties and provide them with the same information. However, another possible limitation was that we did not control for possible bias resulting from the one of the parties not conveying their concerns accurately in order to protect the other party.

### Implications and further research

There are substantial international variations in ACP and a poor understanding of how ACP conversations are conducted in patients’ homes. Future studies should investigate the impact of the environment on ACP conversations, in terms of the possibility of expressing values, preferences and needs; the present findings could be used to identify factors to focus on in other contexts.

The findings of this study suggest a need for conversations about the goal and subject matter of ACP ahead of the ACP conversation, as well as a plan for follow-up. Prospective studies are therefore required to determine the need to prepare patients and their relatives and how to implement the optimal process for ACP. Similarly, a plan for a follow-up conversation with an increased focus on relatives might ensure that they are properly included in developing and revising care plans.

## Conclusions

The patients in this study were invited to discuss ACP issues as part of a palliative-care coordination meeting in their homes, along with their relatives, the cancer nurse, GP and PCT. The healthcare professionals considered that planning for continuity and flexibility were important in their preparation, while the homely atmosphere facilitated a caring approach when sensitive issues were discussed. Alternating between serious, disease-related topics and lighter conversations interspersed with humour was facilitated by the home context. The patients and relatives played an active, host-like role, while healthcare professionals acted like guests; this feature helped to reduce any power imbalance. Various medical, social and personal issues were discussed, but current physical symptoms and future medical treatment dominated. Establishing relationships based on trust and security was emphasized. Some patients had already made well-defined decisions about their future palliative care, whereas others were unable to or chose not to form an opinion. The relatives facilitated the discussions and were a source of significant support to the patients while maintaining a reserved attitude towards their own needs. The healthcare professionals expressed that the ACP conversations represented an essential common reference point and provided a shared understanding of the expected disease trajectory. The home context increased the healthcare professionals’ awareness of the patient as a person as well as of the family relationship.

## Data Availability

No datasets were generated or analysed during the current study.

## Abbreviations

ACP: Advance care planning
GP: General practitioner
PCT: Palliative care team
RQ: Research question
